# Statistical and agent-based modelling of the transmissibility of different SARS-CoV-2 variants in England and impact of different interventions

**DOI:** 10.1098/rsta.2021.0315

**Published:** 2022-10-03

**Authors:** J. Panovska-Griffiths, B. Swallow, R. Hinch, J. Cohen, K. Rosenfeld, R. M. Stuart, L. Ferretti, F. Di Lauro, C. Wymant, A. Izzo, W. Waites, R. Viner, C. Bonell, C. Fraser, D. Klein, C. C. Kerr, The COVID-19 Genomics UK (COG-UK) Consortium

**Affiliations:** ^1^ The Big Data Institute and the Pandemic Sciences Institute, Nuffield Department of Medicine, University of Oxford, Oxford, UK; ^2^ The Queen's College, University of Oxford, Oxford, UK; ^3^ School of Mathematics and Statistics, University of Glasgow, Glasgow, UK; ^4^ Institute for Disease Modeling, Bill and Melinda Gates Foundation, Seattle, WA, USA; ^5^ University of Copenhagen, Copenhagen, Denmark; ^6^ Department of Public Health, Environments & Society, London School of Hygiene and Tropical Medicine, London, UK; ^7^ Department of Computer and Information Sciences, University of Strathclyde, G1 1XH Glasgow, UK; ^8^ UCL Great Ormond St. Institute of Child Health, University College London, London, UK; ^9^ https://www.cogconsortium.uk

**Keywords:** agent-based modelling, multivariate regression modelling, COVID-19

## Abstract

The English SARS-CoV-2 epidemic has been affected by the emergence of new viral variants such as B.1.177, Alpha and Delta, and changing restrictions. We used statistical models and the agent-based model Covasim, in June 2021, to estimate B.1.177 to be 20% more transmissible than the wild type, Alpha to be 50–80% more transmissible than B.1.177 and Delta to be 65–90% more transmissible than Alpha. Using these estimates in Covasim (calibrated 1 September 2020 to 20 June 2021), in June 2021, we found that due to the high transmissibility of Delta, resurgence in infections driven by the Delta variant would not be prevented, but would be strongly reduced by delaying the relaxation of restrictions by one month and with continued vaccination.

This article is part of the theme issue ‘Technical challenges of modelling real-life epidemics and examples of overcoming these’.

## 1. Introduction

**Figure 1.**
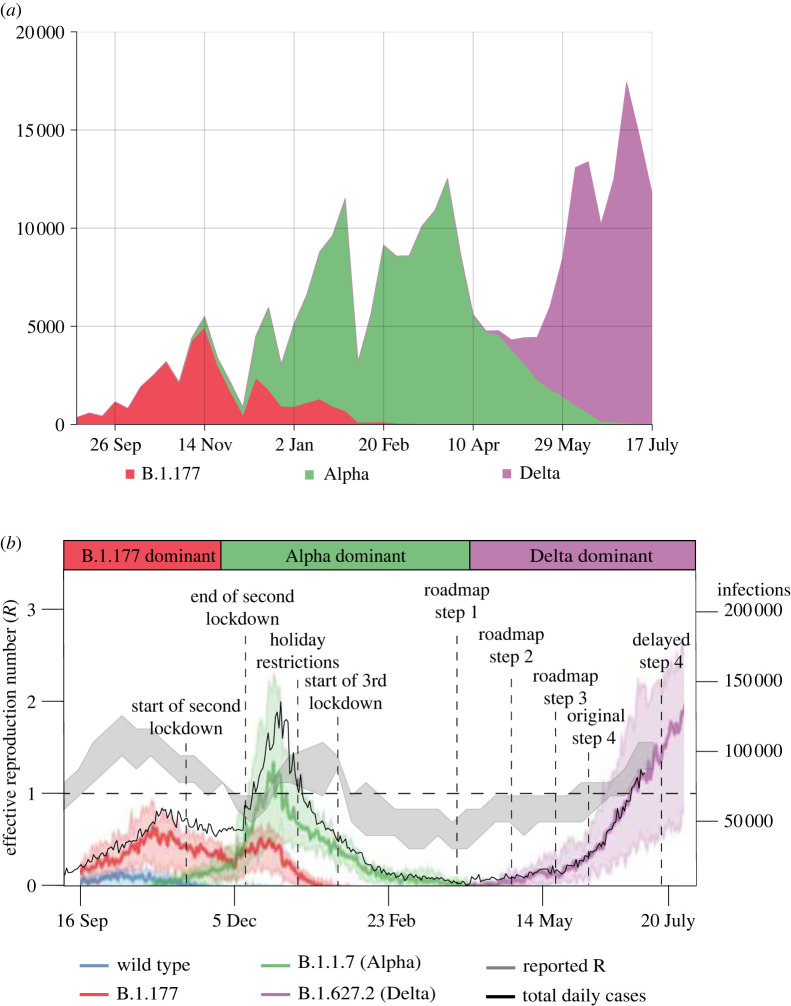
(*a*) The frequency of different SARS-CoV-2 variants using data from the COVID-19 Genomics UK Consortium (COG-UK) [1] as absolute numbers of genomes per week (as a proxy for infections by a circulating variant) and illustrating the consecutive SARS-CoV-2 variants over the study period. (*b*) Covasim model-generated daily infections by different SARS-CoV-2 variant type (blue, red, green and purple lines), together with data on the total number of daily cases (black line; all on the right y-axis) and the reported effective reproduction number R value (grey band and on the left y-axis), in England between September 2021 and June 2021. Bold coloured lines show the median over 100 simulations, and the shaded intervals around these show the 90% CI across the simulations. (Online version in colour.)

Severe acute respiratory syndrome coronavirus 2 (SARS-CoV-2), the virus causing COVID-19, has continued to spread in England throughout 2020 and 2021. Spread was facilitated by the emergence of new viral variants such as B.1.177, B.1.1.7 (Alpha) and B.1.617.2 (Delta), which dominated in late 2020 and early 2021 (figure 1). By July 2021, over 4.2 million confirmed cases and over 122 thousand deaths related to COVID-19 had been reported in England [2].

The first wave of infections in 2020 in England was driven by the wild-type SARS-CoV-2 strain without mutations, which significantly changed the phenotype [3]. It was suppressed by the first national lockdown between March and June 2020 (figure 1); by June 2020, the number of infections caused by the wild-type were declining (figure 1). Other viral variants with different mutations to the phenotype subsequently emerged [1], notably B.1.177 which first appeared in England in late summer 2020 and was the dominating variant over early autumn (figure 1). The Alpha variant or B.1.1.7 was first detected in southeast England at the end of September 2020 and had become the dominant variant by late November 2020 (figure 1). Unlike other strains circulating during autumn 2020, the Alpha variant had spread widely nationally by late December 2020, leading the UK Government to impose a national lockdown from 4 January 2021 [4].

In addition to this third national lockdown, an age-prioritized COVID-19 mass-vaccination programme was deployed across England from December 2020. On 12 July 2021, 80% of the total population in England had received one dose and 61% of the total population had received two doses of vaccine [2].

As a result of the third national lockdown, and the successful vaccination strategy in early 2021, there was a decline in COVID-19 cases and a reduction in the effective reproduction number R below 1 over January to February 2021 (figure 1). In late February, the UK Government therefore announced a ‘Reopening Roadmap’ for 2021 [5] schematically shown in figure 1. The roadmap comprised four consecutive steps of relaxing COVID-19 restrictions alongside a continual mass-vaccination strategy as the planned exit strategy out of the COVID-19 epidemic. Step 1 on the roadmap was the reopening of schools from 8 March 2021 and relaxing of the ‘stay at home rule’ from 29 March 2021. Steps 2 and 3 followed from 12 April 2021 and 17 May 2021 with further restrictions relaxing slowly.

Step 4 on the roadmap, with full relaxation of the social distancing measures under ‘back to normal’ scenario, was originally planned for 21 June 2021. However, during the weeks leading up to this date, concern grew over the safety of relaxing restrictions due to rising rates of infection linked to the Delta variant. The Delta variant or B.1.617.2 was first detected in India and began spreading in England from the middle of April 2021, eventually becoming the dominant variant. There was a notable increase in COVID-19 cases and hospitalizations with COVID-19 ahead of the planned date for step 4: between 23 May 2021 and 17 June 2021, daily reported cases rose from 1734 to 9371 and daily hospitalizations rose from 114 to 242 [2]. (The number of deaths from COVID-19 remained low: 7 on 23 May 2021 and 6 on 17 June 2021.) Previous studies have suggested that the rapid sweep of the Alpha variant from December 2021 was due to it being much more transmissible than previously circulating variants [6,7]. Similarly, the increase in cases and hospitalizations in June 2021 has been attributed to possible higher transmissibility of the Delta variant compared to previous circulating strains [8], although behavioural effects may have also played a role.

Here, we report on analyses performed in June 2021 that were used to help decide whether step 4 on the roadmap should proceed as planned or be postponed. We used statistical analysis of genomic surveillance data [1] and the agent-based model Covasim [9] to evaluate the growth advantage of the B.1.177, Alpha and Delta SARS-CoV-2 variants compared to previously circulating variants in the period between September 2020 and July 2021, and explored the epidemic trajectories in England in the first half of 2021. We illustrated the application of the calibrated Covasim model across different scenarios quantifying the impact of the vaccination strategy and of the Delta variant on planning cases, hospitalizations and deaths alongside the four steps of the roadmap in England in the first half of 2021.

This study extends existing Covasim work by explicitly modelling different SARS-CoV-2 variants, vaccination against COVID-19 in early 2021, and the impact these had on the epidemic in England.

Our results, alongside those of other modelling groups [10] were used to scientifically advise the UK Government to delay step 4 till 19 July 2021. Specifically, our results were discussed at meetings of the Scientific Pandemic Influenza Group for Modelling (SPI-M) and were part of the evidence for the consensus statement on the advised delay of step 4 of the Reopening Roadmap [11].

## 2. Methods

### (a) Statistical analysis to quantify variants transmissibility

To assess the relative transmissibility of different SARS-CoV-2 variants in England, we used publicly available sequencing data from the COG-UK Consortium [1] between September 2020 and 12 July 2021. Multivariate regression analyses quantified the change in the growth rate in Alpha and Delta relative to the previous dominant variant B.1.177, which dominated between September 2020 to November 2020 (figure 1). The count of each variant in each of the 311 lower tier local authorities (LTLAs) was modelled as a function of multivariate smooth functions across latitude, longitude and day.

The variation in spread across variants was tested using a hierarchical generalized additive model (HGAM) following the method in [12]. Generalized additive models (GAMs) have the general regression form:

2.1 $$E(Y)=g^{-1}(\beta_{0}+\sum_{j=1}^{J}f_{j}({\text{x}}_{\text{j}})),$$

where E(Y) is the expected value of the response (count) assuming an appropriate distribution with link function g(.), $\beta_{0}$ is an intercept and $f_{j}$ is a smooth function (often a spline) of covariate(s) $x_{j}$.

In addition to standard fixed effects that are estimated in linear regression models, these non-linear regression models allow for smooth functions of covariates, and interactions between these, allowing for similarities between observations close in space and/or time. Within the regression, the variant can be treated both as a fixed factor (estimating the average transmission rate across the study domain) and/or as a random effect (where the smooth effects over other covariates are estimated separately for each variant). The HGAM approach follows naturally from standard GAMs [13] to allow variation in the smooth relationships across group levels, which in this study are proxies for variants. In this method, the smooth functions are functions of varying combinations of district latitudes, longitudes, date and variant.

The latitude and longitude of each district were taken from ONS data [14]. Districts that are closer together were assumed to be more similar than those further away, so spatial correlation was accounted for using the smooth effects. This can also assist with smoothing out data errors inherent in these observational studies, such as delays in reporting or variations in effort across local areas or time.

We treated each variant as a separate group and fitted a range of different models with a variety of different functional relationships, selecting the preferred model using the Akaike’s information criterion (AIC; further details of the models are in the electronic supplementary material). The AIC penalizes those models that fit the analysed data marginally better but are overparameterized, and therefore are expected to not fit well to other data.

The weekly count of variants in each local district was modelled using a negative-binomial distribution, which allows for potential overdispersion relative to the equal mean-variance relation assumed under a Poisson distribution. The count of each variant across each LTLA and week was the response variable, with a log-link function between linear predictor and response in all model formulations. A variety of different linear predictors were fitted, including fixed effects, smooth effects and random effects. Following [12], we commenced the analysis with the simplest model assuming equivalence between variants and worked up to the most complex model assuming complete segregation between variants. HGAMs have the benefit over generalized linear mixed models, used, for example, in [7], of being able to account for a wider range of non-linear structures in the data, and also of allowing for the possibility of greater forms of variation in trends across and between variants.

We fitted the equivalent of the five models used in [12]. These were built hierarchically based on whether each model had a single smoother or whether they had joint ones across groups (variants), and whether these had the same degree of smoothness (often termed wiggliness). The descriptions of the models fitted can be found in table 1.

**Table 1.** HGAM model descriptions, corresponding to the $f_{j}$ functions in equation (2.1). All models contain the common intercept term, the same log-link function and a negative-binomial distribution for the response variable.

model	description
G	single smooth function of latitude, longitude and week, fixed across variants
I	fixed factor slope for each variant; smooth function of latitude, longitude and week, estimated separately for each variant and with different levels of smoothness
S	smooth function of latitude, longitude and week, estimated separately for each variant, but assumed to have same level of smoothness
GI	as model I, but with additional smooth function of latitude, longitude and week shared across all variants
GS	as model S, but with additional smooth function of latitude, longitude and week shared across all variants

Using the best-fit model, determined by the lowest AIC value, we quantified the average multiplicative advantage of the Alpha variant versus the previously dominating B.1.177 variant and of the Delta variant versus the previously dominating Alpha variant, following a similar approach to [6]. That is, we estimated the difference in exponential growth rates between competing variants Δr, which we transformed into a relative transmissibility value R(t) via R(t)∼exp⁡(ΔrT), where T is the mean generation time which is assumed to be 5.5 days as in [6]. We note that this is an approximate transformation, assuming the generation time T distribution is a delta function concentrated at its mean. A more accurate transformation would be to write $1/R(t)=\int_{\tau =0}^{\infty }w(\tau )\exp(-r\tau )\;d\tau$, where τ is time since infection and w(τ) is the generation time.

To quantify this relative transmissibility value, we compared the model-fitted average trends of each variant over the time period when they started to dominate across LTLAs as a proxy for the relative progressive transmissibility. Specifically, we compared the contrast in temporal trend of the Alpha variant versus the B.1.177 variant over the period between 7 November 2020 and 30 January 2021; and of the Delta variant versus the Alpha variant between 5 May 2021 and 12 July 2021. These time periods were chosen to reflect periods when the emerging variant (Alpha and Delta respectively) were growing exponentially.

All analyses were conducted using the mgcv package [15] in R with the numerical code freely available at https://github.com/Jasminapg/PTRSA-Covasim-paper .

### (b) Dynamic modelling of the COVID-19 epidemic using Covasim

During 2020 and 2021, the stochastic agent-based model Covasim [9] has been widely used across a number of settings to track the status of the COVID-19 epidemic and to explore the impact of different non-pharmaceutical and pharmaceutical interventions. For example, in the UK, Covasim has been used to evaluate the impact of different Test-Trace-Isolate strategies when schools reopened in the UK after the first national lockdown [16], to explore the impact of wearing face coverings in schools in the second half of 2020 [17] and to simulate different scenarios of schools’ reopening at step 1 of the roadmap [18]. In addition, within the UK Health Security Agency, Covasim is used for nowcasting epidemic metrics such as the reproduction number R and the growth rate r that track the status of the COVID-19 epidemic [19]. Internationally, Covasim has been applied in settings within the USA [20], used to inform decision-making in Australia [21] and used to inform the impact of reopening borders in Vietnam [22], with a number of other studies ongoing.

In this study, we extended Covasim to allow co-circulation of multiple viral variants, allowing us to model B.1.177, Alpha and Delta in England over the period September 2020–June 2021 and also incorporated age-prioritized vaccination against COVID-19. Details of the Covasim’s core modelling framework have been published [9]; we briefly review these in §2bi. The specifics of modelling different variants and vaccination are contained in §2bii and 2biii with further details on modelling vaccination in [23]. We used social mobility data to model behaviour changes, with details in §2biv, and simulated different non-pharmaceutical interventions as described in §2bv.

#### (i) Covasim model

**Figure 2.**
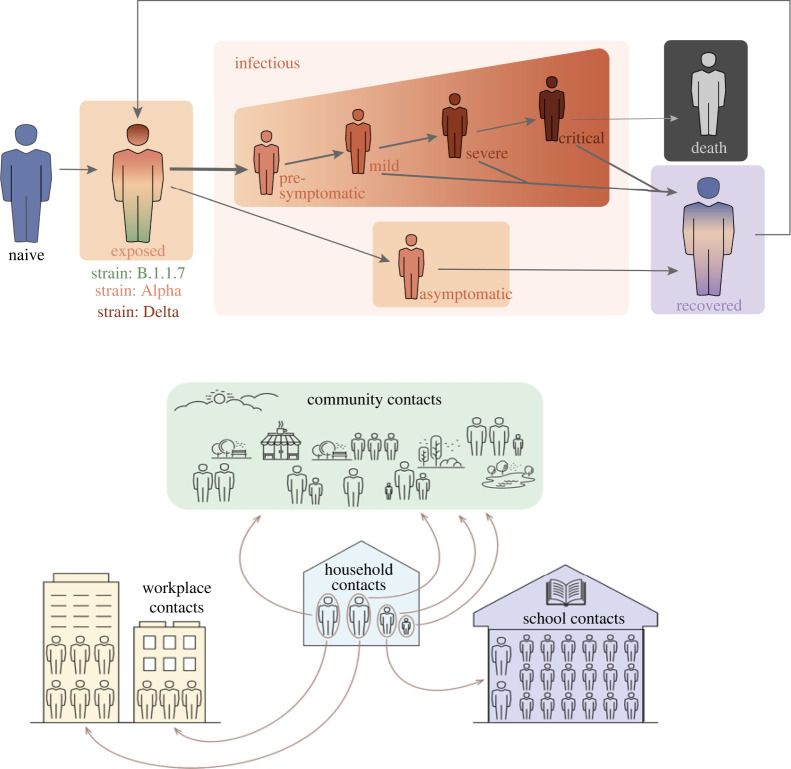
Schematic of the Covasim model showing the different modelled layers within society. This figure is reproduced with permission from [9]. (Online version in colour.)

In Covasim, individuals susceptible to SARS-CoV-2 infection move through an exposed stage and infectious stages (asymptomatic, presymptomatic, mild, severe or critical) of infection, before either recovering or dying (figure 2*b*). Recovered individuals can later lose their immunity and become reinfected. The model also incorporates heterogeneity in infectiousness with age. The underlying structure is ‘SEIRS’ (susceptible-exposed-infectious-recovered-susceptible) with specific parameters describing the cross-immunity between the different variants modelled.

In this study, we used Covasim’s default parameters and the ‘hybrid’ network structure for England (see §2.4 of [9] for details), with the default data included in the model for English population age structure and household sizes. The ‘hybrid’ approach bridges between a fully random network and a fully data-derived network with individuals in the population modelled to have contacts in their household, school (for children), workplace (for adults) and community. In this study, the population was stratified across four population contact-network layers: schools, workplaces, households and community settings (figure 2) with pre-defined contact patterns across these layers based on the Polymod study [24]. Within this framework home, school and work contacts are repeated, while those in the community layer are not. We also note that there is an overlap between community contacts with home, school and work layers, allowing people to fall in more than one layer and reflective of reality. A population of individuals was generated according to a location-specific age distribution—in this case England—and each individual was randomly assigned to a household using England-specific data on household sizes. Children were assigned to schools and adults to workplaces, each with a user-specified number of fixed daily contacts (by default, Poisson distributed with means of 20 for schools and 16 for workplaces). Individuals additionally were modelled to have contacts with others in the community which are Poisson distributed with a mean that reflected the stage of the epidemic and varies depending whether there is a lockdown, reduced mixing or pre-COVID-19 mixing levels.

In this study, using Covasim v. 3.0.7, we generated a population of 100 000 agents interacting over the four contact-network layers (households, workplaces, schools and communities). Reflective of the size of the population of England, we used dynamic scaling of these 100 000 agents up to the population of around 56 million. Dynamic scaling is a standard approach within individual-based-models such as Covasim, which allows for arbitrarily large populations to be modelled whilst maintaining a constant level of precision and manageable computation time throughout [9].

#### (ii) Modelling different SARS-CoV-2 variants

In our previous work, we either modelled a single strain (wild type) of SARS-CoV-2 [16,17] or implicitly modelled the effect of two strains (wild type and Alpha variant) [18]. In the latter case, we simulated a single strain with time-varying infectiousness, including a logistic growth function for the relative proportion of the Alpha variant from 1 September 2020 and estimated the increased infectiousness of Alpha compared to the wild type by fitting to the increased growth in cases, in an approach similar to other work [6].

In this study, in contrast, we use new features in Covasim to mechanistically model individual SARS-CoV-2 variants by allowing different model parameters to be introduced to characterize each variant. Details of this will be explored in a separate study; briefly, we have modelled three different variants (figure 1) circulating in England in 2020 and 2021: the wild type of SARS-CoV-2 which emerged in early 2020, the B.177 variant which emerged in August 2020, the Alpha variant which emerged in late September 2020 and spread nationally between October 2020 and February 2021, and the Delta variant which emerged in late April 2021 and became the dominant variant.

The parameters characterizing these variants are (a) the number of imported or seeded infections for each emerging variant at the time of emergence, (b) the relative transmissibility of each variant, (c) vaccine effectiveness against each variant and (d) the cross-immunity between variants.

**Figure 3.**
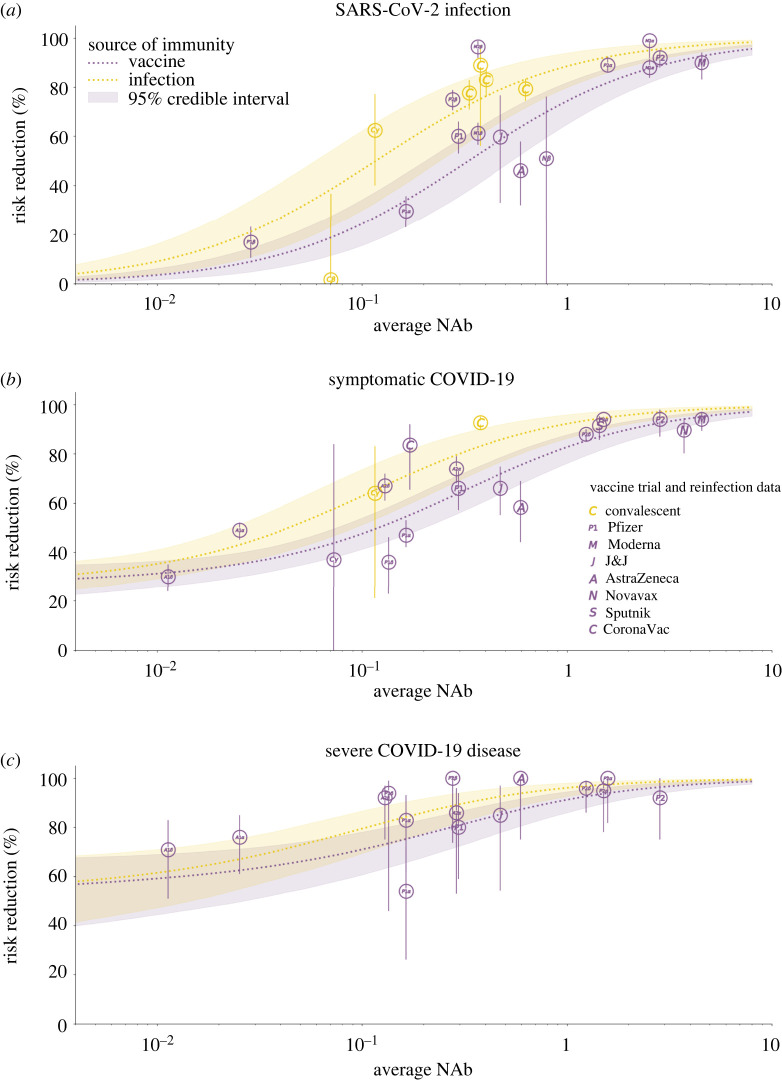
Modelled vaccine efficacy used in the Covasim model for this study. Average NAb refers to the cohort-average NAb level for each individual study (represented here as a bubble), normalized to be relative to convalescent NAbs. The risk reduction in infection, symptomatic COVID-19 disease and severe COVID-19 disease following vaccination or infection are modelled as functions of NAb level. Details of the method and data sources used are given in [23]. (Online version in colour.)

In this analysis, the number of imported cases and transmissibility for each variant were determined during the calibration process from within the ranges derived in the statistical analysis. The assumed effectiveness of vaccines against each variant is shown in figure 3 and discussed further in the next section. We explicitly modelled the ability of variants to escape immunity from prior infection or vaccination using data from binding neutralization studies [25].

#### (iii) Modelling COVID-19 vaccination

We modelled an age-prioritized vaccination schedule with two doses given 8–12 weeks apart. Vaccines provide partial protection against SARS-CoV-2 infection, reduce disease severity and reduce onward transmission. The schedule we used reflects the vaccination in place in England in July 2021, namely the Pfizer/BioNTech vaccine for individuals aged 65+ or under 40, and the Oxford/AstraZeneca vaccine for individuals aged 40–64. In July 2021, the strategy was to vaccinate all adults (18+ years old) with one dose by 31 July 2021, and with a second dose within 12 weeks of the first. We did not include vaccination of those under 18 years apart from a small proportion (10%) of this cohort with underlying conditions which would have been vaccinated under high-risk groups. Follow-up work will evaluate the impact of future inclusion of the vaccination of those under 18 years.

Vaccine efficacy is modelled through an immune response that primes and boosts neutralizing antibodies (NAbs) in individuals and then relates the level of NAbs to protection against infection, symptomatic disease and severe disease [23,26,27]. The model accounts for waning NAbs over time and has been fit to vaccine efficacy and effectiveness from data in trials to date (figure 3). For this study, we model the reported immune response induced by the Pfizer/BioNTech and Oxford/AstraZeneca vaccines as well as the ability of the Delta variant to evade vaccine- and infection-derived immunity, which results in efficacy values within the reported ranges of these vaccines’ efficacy against Alpha and Delta [28].

#### (iv) Modelling behaviour change

Within the model, we incorporated a dynamic transmission probability to be reflective of changes in the social mobility within different layers of society, updated weekly (figure 2). Overall levels were reflective of reported Google mobility changes [29], but scaled at different times to reflect changes such as school holidays, national lockdowns and more gradual social mobility increase during the phased reopening from March 2021 as in other studies [30]. These were necessary since the mobility changes in the Google reports stratify society in different ways to how we stratify society in the layers depicted in figure 2. Next we elaborate on these.

Within the school layer, we simulated children of different ages as attending either primary or secondary schools, with attendance changing due to the three COVID-19-related lockdowns and school holidays. During the third national lockdown children of key workers attended school, with estimates suggesting that around 20% of primary school students and 5% of secondary schools students were attending—an average of 14% of children [31]. For simplicity, we included this in the modelling framework by simulating transmission intensity at 14% of the expected level at full attendance between 4 January 2021 and 8 March 2021. When schools reopened from 8 March 2021, we assumed a reduction in the per-contact transmission probabilities by 37% in schools, i.e. simulated 63% of transmission within schools remaining from September. This was modelled as an aggregated reduction in transmission due to hygiene, mask usage and other social distancing measures in place within schools to reduce transmission, and as described in detail in [17].

For workplace social mixing, we also used Google mobility data to obtain a broad range of the change over time. Specifically, as in our previously published studies, we simulated workplace and community transmission to be 20% of their pre-COVID-19 levels during the first lockdown in 2020 and 30% during the second and third lockdown, increasing to 40% from step 2, i.e. 12 April 2021, to 50% from step 3 (17 May 2021) and going back to 70% in workplaces and 70% in community from step 4 in either June or July 2021.

We modelled household transmission intensity in line with the average monthly level of increased household mobility in the Google data [29].

#### (v) Modelling non-pharmaceutical intervention

In Covasim, testing is modelled using parameters that determine the probabilities with which people with different symptoms receive a test each day, for both symptomatic and asymptomatic people, based on the reported testing levels from [2]. We note that the symptomatic testing rates were assumed constant for the post-step 4 period. Tracing is modelled using parameters for the probability of reaching the contacts of those testing positive, as well as the time taken to reach them. We assume that some layers of society would be easier to trace than others; specifically, that 100% of household contacts can be traced within the same day, 80% of school and workplaces can be traced within 1 day and 10% of community contacts can successfully be traced within 2 days. This results in an average of 60% of contacts traced across different layers between January and May 2021, comparable with reported monthly values from [32]. The level of adherence to isolation has parameters that differ across different layers of the population (see data sources and calibration section below). Following discussions with NHS Test & Trace, we modelled 60% efficacy of isolation over the period January 2021 and May 2021.

#### (vi) Modelling hospitalizations

In Covasim, hospital admissions are modelled by the number of severe cases. During the calibration process, the time series of severe cases were matched to the data on daily hospital admissions from the COVID-19 Dashboard by deriving an optimal value of a parameter that transferred from diagnoses to severe cases.

### (c) Data sources and calibration

For the regression analysis we used publicly available COVID-19 Genomic Surveillance data from [1]. The data are aggregates of genomes sequenced at the Wellcome Sanger Institute to monitor COVID-19 dynamics. The number of each lineage is enumerated by week and local authority, with dates corresponding to the date on which the sample was collected.

For the epidemic trajectories, data from the UK-COVID-19 Dashboard [2] were extracted consisting of daily measurements of reported cases, deaths and hospitalizations between 1 September 2020 and 20 June 2021.

We fixed the model parameters at point estimates over the period 20 January 2020 to 1 September 2020 based on values we used in our previous study [16] to fit metrics of the epidemic during this time in England. We then calibrated the model to data from the UK-COVID-19 dashboard over the period 1 September 2020 and 20 June 2021 using Optuna’s [33]’s parameter sweep to find optimal values for nine separate parameters: (a) the number of seeded infections of the three variants of concern (B.1.177 in September 2020, of Alpha in October 2020 and of Delta in mid-April 2021); (b) the relative transmissibility of each of the three variants compared to previous circulating variants and (c) the average monthly symptomatic testing rates in each of the 3 months from March to May 2021 inclusive. The calibration process was done using the Optuna [33] hyperparameter optimization framework in Python to search the nine-dimensional parameter space for optimal values that minimized the absolute difference between the model’s estimates of daily cases, deaths and severe infections (representing hospitalizations), and the corresponding data on cumulative and daily infections by date reported, deaths within 28 days, and admissions to hospital by date reported between 1 September 2020 and 20 June 2021.

By using the calibrated model, and v. 3.0.7 of Covasim, we ran 100 simulations and generated the median of the simulations as the central estimate as well as the 25th and 75th percentiles comprising the confidence intervals (CIs) of the simulations. Across the different scenarios, we projected these for the daily new diagnoses with COVID-19, daily number of severe COVID-19 cases (as a proxy for hospitalizations) and daily deaths from COVID-19 over the period 1 September 2020 to 31 August 2021.

The numerical code and the data used to generate the projections across the scenarios reported in this paper are available at https://github.com/Jasminapg/PTRSA-Covasim-paper.

## 3. Results

### (a) Results from the statistical analysis

Table 2 contains the model comparison results of the statistical analyses of the COG-UK dataset. Model GI had the lowest AIC value and hence was used for inference. Significance of smooth terms was calculated by the mgcv package according to the approach of [34], using Wald-type tests with null hypothesis being that the smooth function is exactly zero. All smooth terms were highly statistically significant at the p=0.05 level, calculated according to the approach of [34] ($p<2\times 10^{-16}$ in all cases). Absolute model fit was tested by comparing observed and model-fitted values (correlation 0.8) and using residual checks. These can been found in the supporting information and imply that there are sufficient differences between the variants that models incorporating variant-specific smooth terms are required for accurate analysis. The multivariate smooth terms of latitude, longitude and day were highly statistically significant (both global and variant-specific), whereas variant-specific factor slopes were not, suggesting that simple models fitting general trends across all regions are not sufficiently flexible. This is further supported by the fact that model I had the second lowest AIC across the fitted models.

**Table 2.** ΔAIC values and effective degrees of freedom (EDF) for the different HGAM formulations, that is the difference in AIC between each of the models and the best-fitting model (i.e. a value of zero corresponds to the preferred model).

model	ΔAIC	EDF
G	10330.2	399.8
I	908.7	859.3
S	1553.2	615.8
GI	0	980.9
GS	1569.3	673.4

**Figure 4.**
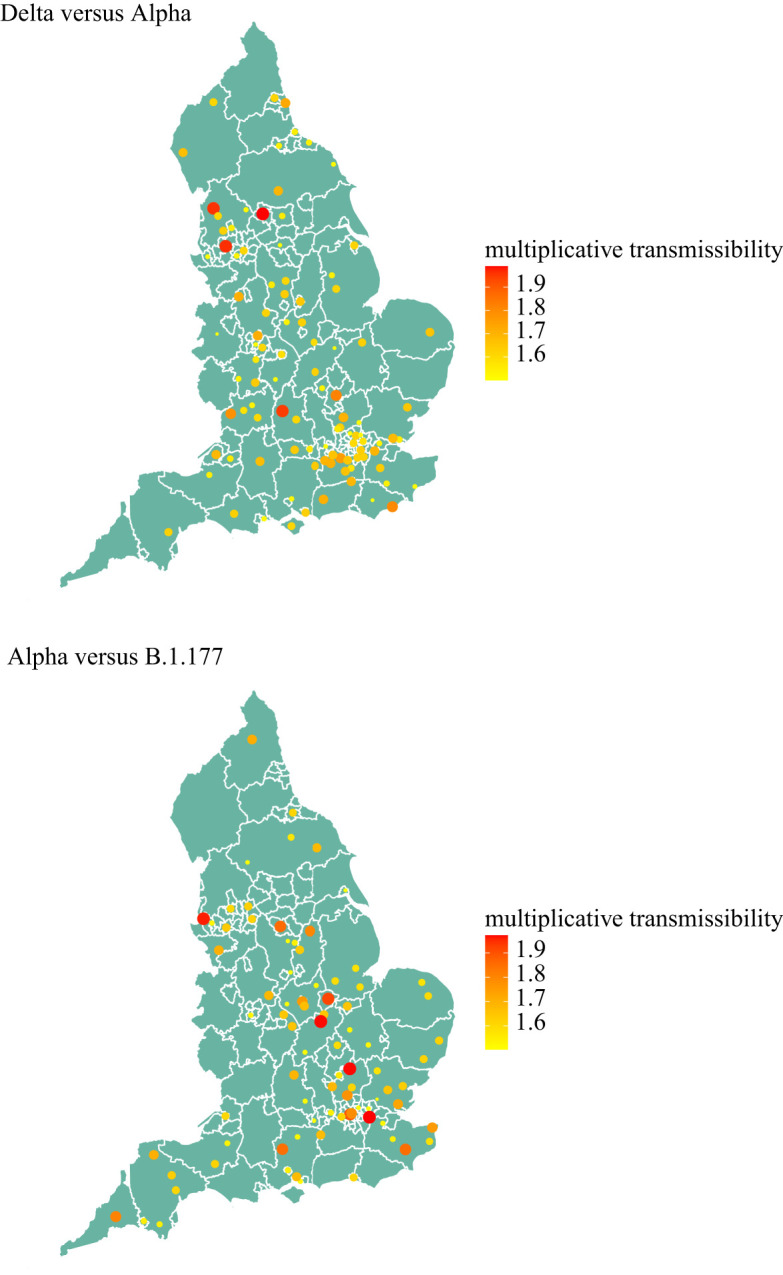
Relative transmissibility of Delta to Alpha variant (top plot) and Alpha variant to variant B.1.177 (bottom plot) across LTLAs for the period 5 May to 12 July 2021 (Delta versus Alpha), and 7 November 2020 to 30 January 2021 (Alpha versus B.1.177). Only LTLAs with transmissibility greater than 50% higher are shown. (Online version in colour.)

Our results suggest that the relative growth of Delta was higher than that of the previously circulating strain Alpha, and hence, Delta was able to outcompete other SARS-CoV-2 strains in England since May 2021 (figure 4 (top plot)). This is a similar pattern of behaviour to the Alpha variant, which outcompeted the previously dominating variant B.1.177 and was the dominating variant in late 2020 and early 2021, including B.1.177 (figure 4 (bottom plot)).

The observed transmissibility of the variants across England was spatially heterogeneous (figure 4) but Delta was consistently more transmissible than Alpha, while Alpha was consistently more transmissible than B.1.177. In LTLAs with very large (over 50%) and consistent occurrence of the Delta variant, transmissibility of Delta was 65–90% greater than Alpha, but spatially heterogeneous (figure 4 (top plot)). Similarly, the estimates for Alpha being more transmissible than B.1.177 varied between 50 and 80% across spatial regions (figure 4 (bottom plot)).

### (b) Modelling COVID-19 epidemic dynamics in England with Covasim

The model’s estimates of cases, hospitalizations and deaths were consistent with increased transmissibility of the B.1.177, Alpha and Delta variants relative to previous circulating variants. Analogous to the statistical analysis, Delta had the highest transmissibility amongst all the circulating variants and hence has been dominating in England since May 2021 (figure 1). We found that the reported counts of cases, hospitalizations and deaths were consistent with model estimates in which the relative transmissibilities were 1.2 ((95%CI=[1.14,1.27])) for B.1.177, 1.8 (95%CI=[1.59,1.86]) for Alpha and 2.6 (95%CI=[2.52,2.71]) for Delta. This would imply that, on average, B.1.177 was 20% more transmissible that the dominating variants circulating in England during September 2021, Alpha was 60% more transmissible than B.1.177 and Delta was 80% more transmissible than Alpha.

**Figure 5.**
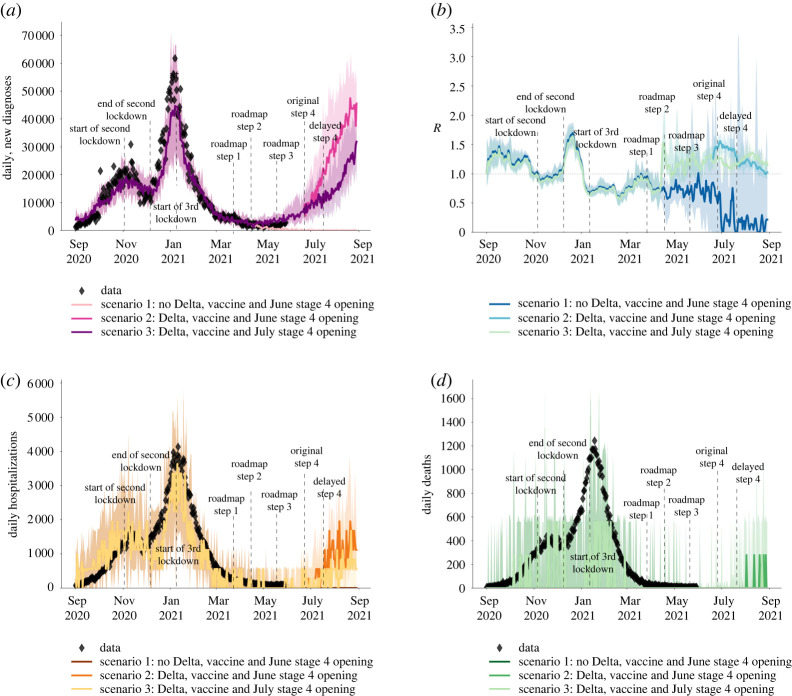
The modelled impact of delaying step 4 of the roadmap by one month with and without the spread of the Delta variant, showing (*a*) the number of cases, (*b*) the effective reproduction number, (*c*) hospitalizations due to COVID-19 and (*d*) deaths related to COVID-19. The data from [2] are shown in diamond shapes, with the model-generated simulations overlayed. (Online version in colour.)

**Figure 6.**
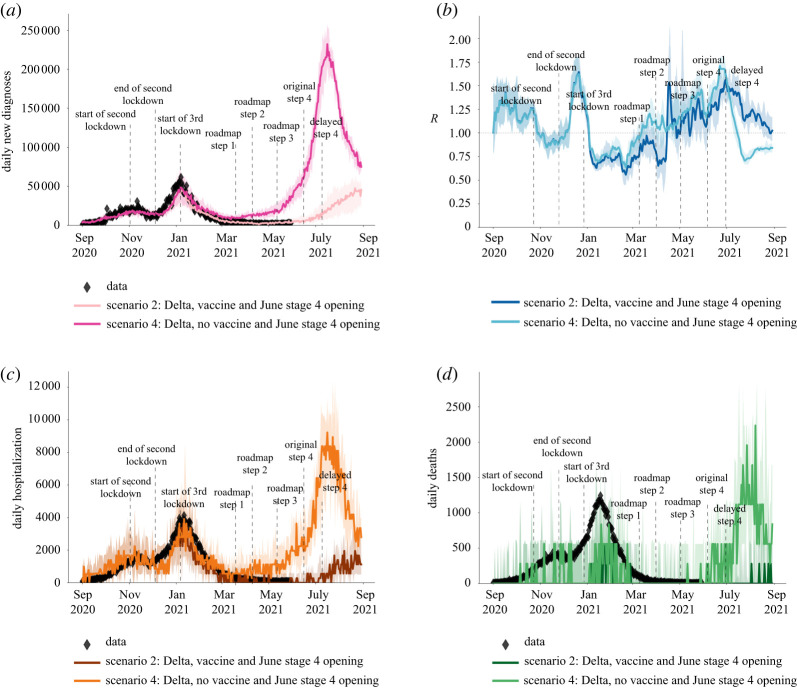
The modelled impact of vaccination against COVID-19 over the first half of 2021, showing (*a*) the number of cases,(*b*) the effective reproduction number, (*c*) hospitalizations due to COVID-19, and (*d*) deaths related to COVID-19. The data from [2] is shown in diamond shapes, with the model-generated simulations overlayed. The two curves in each of the subplots illustrate the importance of vaccination in preventing surge in cases, hospitalizations and deaths, as well as an increase in effective reproduction number *R* in early 2021. (Online version in colour.)

Using these values for the relative transmissibility of variants, the model can effectively reproduce the epidemic trajectories of SARS-CoV-2 in England between 1 September 2020 and 20 June 2021 (figure 5), also stratifying the number of daily infections by variant type (figure 1). Our simulations capture the increase in the epidemic as B.1.177, and Alpha variants started to spread from September 2020, with Alpha coming to dominate by the end of 2020. The third national lockdown during January and February 2021 and the vaccination programme which started in December 2020 were successful in suppressing the spread of Alpha and reduced the effective reproduction number below 1 in early 2021 (figures 5 and 6).

Before Delta started to spread nationally, there was a period of epidemic decline with low numbers of cases, hospitalizations and deaths over the period between January 2021 and end of May 2021 (figure 5). This allowed the phased reopening with the steps 1–3 of the roadmap on 8 March 2021, 12 April 2021 and 19 May 2021 to occur.

### (c) Impact of delaying the roadmap by one month

Our results, reported in June 2021 and shown in figure 5*a*–*d* illustrate the importance of Delta in continuing the epidemic spread in England after step 3 of the roadmap, and the importance of delaying the step 4 of the roadmap in preventing a surge in cases, hospitalizations and deaths, as well as an increase in effective reproduction number R in July 2021. After step 3 of the roadmap, with the emergence of the highly transmissible Delta variant from mid-April 2021, the number of cases, hospitalizations and deaths started to increase despite ongoing vaccination (figure 5*a*–*d*). If step 4 had proceeded on 21 June 2021 as originally planned, it would have resulted in a large third wave not only in cases but also in hospitalizations and deaths (figure 5) as well as an increase in the reproduction number over 1, suggesting a rapidly growing epidemic (figure 5*b*). Delaying step 4 by one month was estimated to substantially dampen such a third wave (figure 5). The forecasted increase in cases without a delay in step 3 is larger than the forecasted increase in hospitalizations (figure 5*c*) or in deaths (figure 5*d*).

Overall, we show that delaying the Roadmap Reopening by one month, in June 2021, was expected to significantly dampen a resurgence in infections and hospitalizations, whilst also reducing deaths, though not prevent epidemic growth completely.

### (d) Impact of vaccination in presence of Delta

Our results show that the third national lockdown and the phased reopening in early 2021 was able to suppress the spread of the Alpha variant with the epidemic metrics declining through February, March and April 2021 (figure 6). However, vaccination was necessary to control the spread of Delta from mid-April 2021, with our results highlighting that a very large third wave in cases, hospitalizations and deaths would have been caused by Delta had vaccination been absent in early 2021 (figure 6). With the modelled age-prioritized vaccination programme, the peak of the Delta-driven epidemic wave across all three metrics was predicted to be reduced five times and notably delayed allowing the phased reopening at steps 2 and 3 to start from a lower epidemic level (figure 6*a*–*d*).

## 4. Conclusion

Mathematical and statistical analyses have been widely used to better understand the data and inform the science of the SARS-CoV-2 epidemic. In this article, we used these tools to simulate the spread of different SARS-CoV-2 variants in England between September 2020 and July 2021, evaluating the transmissibility of different variants relative to previously circulating ones and modelling the impact of delaying step 4 of the roadmap in presence of Delta and with continual vaccination.

Both our statistical and our mathematical modelling confirmed that the emerging variants have been progressively more transmissible, with B.1.177 20% transmissible than previous prevailing variants, Alpha 50–80% more transmissible than B.1.177 and Delta 65–90% more transmissible than Alpha. The estimated relative advantage in transmissibility of Alpha over B.1.177 and of Delta over Alpha from the statistical analysis was spatially heterogeneous, but the ranges agreed with the values determined by Covasim. They also agree with previous estimates, for example the transmissibility of Alpha relative to previous circulating variants was estimated as 43–90% by Davies *et al.* [6] and 50–100% by Volz *et al.* [7]. The transmissibility of the Delta variant versus previous prevalent variants is also close to the reported range of 69–83% by Sonabend *et al.* [8].

In agreement with other modelling results [10], our findings also confirmed that the emergence of the highly transmissible Delta variant resulted in a need to delay step 4 of the ‘Reopening Roadmap’ for relaxing restrictions’. These results were used in June 2021 to offer scientific information of the expected consequences of delaying step 4 till July 19 2021.

A novel feature of this work is that, whilst previous studies have shown the relative transmissibility of two simultaneously circulating variants [6–8], this study is the first study to model the sequential competition of more than two variants over different time periods. Both the statistical analysis and Covasim were able to quantify this progressive transmissibility. Understanding competition between variants is crucial for planning responses to future emerging SARS-CoV-2 variants, and the methods presented here can readily be used to study this. For example, in future work, we intend to explore co-infections with different strains of SARS-CoV-2 and different influenza strains. In addition, our analysis estimated the relative transmissibility of different variants using two different approaches: statistical modelling and mathematical modelling. This provided a robustness check within the same study and generated results which are also aligned with previous studies [6–8]. Modelling has been very popular during this pandemic, and while a number of separate statistical and mathematical models have been used for inference and for nowcasting or forecasting under different scenarios, statistical and mathematical modelling have only rarely been used in parallel to untangle the same question, with some exceptions [35].

We note that Covasim is a stochastic model and as with any stochastic modelling, there is uncertainty in predicted outcomes arising from inherent stochasticity, in addition to uncertainty about the values of parameters controlling the process. The uncertainty in our predictions can be seen in figures 5 and 6. Our results were based on taking the median of 100 simulations from a stochastic process. This uncertainty increases when predictions are made over a longer time period. Hence, in figures 5 and 6, we only projected for 4 weeks into the future. Projecting results of any model, including ours, too far into the future based on current data is unwise due to increasing uncertainty (including uncertainty about the continued validity of model assumptions). Importantly, estimation of future epidemic trajectories beyond July 2021 will depend on the effect of expanding vaccination to include individuals younger than 18 years, and also any third dose boosters to the older population cohorts, and we will explore these in future studies.

Our analyses have illustrated the importance of delaying the step 4 of the roadmap in England to reduce its impact on the surge of epidemic. Appropriate timing of full reduction of the COVID-19 restrictions in conjunction with the fast immunization programme against COVID-19 have been essential in preventing large surges in the English epidemic, and this should be a lesson to other countries as they are faced with exit strategies out of lockdowns.

Our work has some limitations and aspects that require further study. Firstly, our statistical approach used the latitude and longitude of LTLAs to assume a smoothed Euclidian representation of distance across England. This may not be the most appropriate representation for infectious diseases that have varying means of transmission across space, such as transport networks or household/employment networks, and alternative covariates could be chosen to determine links between neighbouring regions. The impact of this choice of spatial distance may be limited when aggregating to a larger spatial scale such as LTLA, compared to 1 km grid squares, for example, and this may be a reason for the spatial heterogeneity in the observed transmission rates in this study. Future work will explore this in more detail combined with the extended statistical analysis, for example looking at different timeperiods.

Secondly, in this study and when using Covasim in general, though we have aimed to use the most recent data from the literature to parameterise the model, some questions remain unanswered. For example, we collated evidence to develop a separate vaccine effectiveness model, detailed in [23]. However, vaccine efficacy against onward transmission of the Delta variant and waning protection from both vaccination and infection are uncertain from current data. Specifically, the version of Covasim used in this study assumed a single antibody waning function for all individuals and all types of immunities, with individual- and immune-level variation in the level of NAbs. As future results reveal differences in the antibody kinetics of infection- vs. vaccine-derived NAbs, this assumption will be revisited. In addition, Covasim is being adapted to capture the effect of heterologous immune sources, including different vaccines administered at different intervals. This topic will continue to grow in relevance as countries roll-out vaccine boosters to their populations, and we plan to revisit it in future work. Furthermore, we note short-term epidemic dynamics are driven by assumptions regarding waning immunity and population heterogeneity, which together determine whether a given variant will die out or reach an endemic state. Long-term dynamics, in both Covasim and the real world, are driven primarily by the introduction of new immune-evading variants. Exploring aspects like these is part of our planned future work but out of scope for this article.

Thirdly, we calibrated the model to aggregated English national data on reported cases, hospitalizations and deaths. Given that the statistical analysis suggests a significant variation in the relative growth advantage for successive variants across LTLAs (e.g. 65–90% higher transmissibility of Delta than Alpha across LTLAs with >50% Delta cases), this may reflect local heterogeneity in the epidemic. Ongoing work is extending the England Covasim model across the other three UK nations, as well as across the English regions aiming to capture the epidemic spatial heterogeneity. Future studies will also look at fitting more granular distribution of these epidemic metrics across different ages, especially when modelling the impact of different vaccination strategies including immunization of those younger than 18 years and booster vaccination for elderly people.

Finally, we chose the dates to compare different variants to reflect the period of exponential growth of the emerging variant as per figure 1, and we only considered LTLAs where the prevalence of the variant reached greater than 50%. This method of temporal truncation is different to the method of [7] in which the transmissibilities of Alpha and B.1.177 were compared only over the first 31 days of the emergence of the new variant. In the future work, we will explore the differences in transmissibility when different periods of studies are used and different prevalence threshold values for transmission.

In summary, we used statistical analysis and agent-based modelling to quantify the progressive transmissibility of three SARS-CoV-2 variants that have been circulating in England between September 2020 and July 2021 and used this information to model the epidemic in England over this period. Our findings confirm that each new variant became progressively more infectious. The emergence of highly infectious Delta variant in late spring 2021 resulted in a need for a one-month delay of step 4 of England’s COVID-19 roadmap out of lockdown. In our simulations, conducted in June 2021, we found that the delay could not prevent but could drastically dampen the projected resurgence due to the Delta variant, which was further suppressed as a result of the age-prioritized vaccination programme from December 2020.

## Data Availability

Data used in this paper are publicly available at https://covid19.sanger.ac.uk/lineages/raw and at https://coronavirus.data.gov.uk. Electronic supplementary material is available online [36].
